# 874. General Surgery vs Plastic Surgery Departments' Publication Output in Burns Literature

**DOI:** 10.1093/jbcr/irag033.351

**Published:** 2026-04-06

**Authors:** Francesco M Egro, Kian Daneshi, Sarah M Tepe, Christopher J Fedor, Mare G Kaulakis

**Affiliations:** University of Pittsburgh Medical Center, Pittsburgh, PA; School of Population Health and Medicine, University of Sheffield, London, England; University of Pittsburgh Medical Center, Pittsburgh, PA; University of Pittsburgh Medical Center, Department of Plastic Surgery, Pittsburgh, PA; University of Pittsburgh Medical Center, Mercy Burn Center, Pittsburgh, PA

## Abstract

**Introduction:**

Both plastic surgeons and general surgeons are involved in the treatment of burn patients; however, their relative contributions to the academic burn literature remain unclear. Prior studies have examined authorship trends in plastic surgery, but none have specifically compared output between surgical specialties in the field of burns. Understanding which specialties are driving burn-related research may provide insight into academic leadership and opportunities for greater collaboration. This study aims to analyze and compare the volume of burn-related publications originating from departments of plastic surgery versus general surgery.

**Methods:**

A total of 18 275 articles were scraped from 12 major burn journals (1982-2025) using the PubMed Entrez API. For string parsing, systematic regex pattern matching was utilized to identify affiliated terms to general surgery or plastic surgery. Statistical analysis included chi-square tests for independence, Wilson confidence intervals for proportions, temporal trend analysis, and effect size calculations using Cohen's w.

**Results:**

Of 16 787 articles with identifiable affiliations, plastic surgery departments participated in 3455 papers (20.6%, 95% CI: 20.0%-21.2%) while general surgery departments participated in 3244 papers (19.3%, 95% CI: 18.7%-19.9%). Inter-departmental collaboration was substantial, with 782 papers (4.7%) involving both departments simultaneously. The involvement ratio revealed minimal difference (1.07:1, plastic:general), with borderline statistical significance (χ^2^ = 6.6, *p*=.010). Effect size analysis indicated negligible practical difference (Cohen's w = 0.020). Temporal analysis demonstrated evolving collaborative patterns across decades, with stable plastic surgery involvement trending from 50.5% (1980s) to 49.8% (2020s), indicating sustained balanced participation rather than specialty dominance.

**Conclusions:**

Burn surgery research is currently shared between general and plastic surgery–trained burn surgeons. While plastic surgery maintains a slight numerical edge, the difference is of minimal practical significance, and long-term trends indicate sustained balanced participation rather than specialty dominance. These trends carry important implications for future research funding priorities, the design of surgical training pathways, and a more comprehensive understanding of the evolving academic landscape in burn surgery.

**Applicability of Research to Practice:**

Both general and plastic surgeons can be trained in burn surgery. The findings of this study note the importance of both specialties in the care of burn patients.

**Funding for the study:**

N/A.

